# Vaginal endometriosis following uterine transplantation in a patient with Mayer–Rokitansky–Küster–Hauser syndrome: A unique case report

**DOI:** 10.1002/ijgo.70531

**Published:** 2025-09-10

**Authors:** Jana Moravcova, Roman Chmel, Jana Maluskova, Eva Sticova

**Affiliations:** ^1^ Clinical and Transplant Pathology Center Institute for Clinical and Experimental Medicine Prague Czech Republic; ^2^ Department of Obstetrics and Gynecology, Second Faculty of Medicine Charles University and Motol University Hospital Prague Czech Republic; ^3^ Pathology Department, Third Faculty of Medicine Charles University and University Hospital Kralovske Vinohrady Prague Czech Republic

**Keywords:** ectopic endometrium, Mayer–Rokitansky–Küster–Hauser, neovagina, transplant complications, uterine transplantation, vaginal endometriosis

Endometriosis is an estrogen‐dependent condition characterized by the presence of endometrial‐like tissue outside the uterus, most frequently involving the ovaries, uterosacral ligaments, and rectovaginal septum.^1^ Vaginal involvement usually occurs in the setting of deep infiltrating disease or following prior surgical intervention.^2^

Mayer–Rokitansky–Küster–Hauser (MRKH) syndrome is a congenital malformation that manifests as uterovaginal agenesis.^3^ Uterine transplantation (UTx) has emerged as a reproductive option for women with absolute uterine factor infertility (AUFI), including those with MRKH syndrome.^4^

In the present study, we report a rare case of vaginal endometriosis in a woman with a neovagina after UTx. To our knowledge, this is the first documented case of endometriosis occurring in the setting of UTx.

A 23‐year‐old woman with MRKH syndrome was referred for UTx. Suspicion of uterovaginal agenesis was raised at the age of 15 due to primary amenorrhea. Magnetic resonance imaging confirmed the absence of a uterus, demonstrating small uterine remnants with no evidence of functional endometrium. In 2012, the patient underwent laparoscopic assisted neovagina creation using the modified Vecchietti technique.^5^ In 2017, she received a uterine graft from her 47‐year‐old mother,^6^ who showed no intraoperative or histologic evidence of endometriosis. The postoperative course was uneventful, with no rejection observed on surveillance biopsies. Immunosuppression was maintained with a triple‐drug regimen consisting of prednisolone, tacrolimus, and azathioprine.

Following transplantation, the patient reported regular menstruation of normal intensity, and endometrial biopsy was not deemed necessary until uterine graft explantation.

Two months after transplantation, the patient experienced progressive narrowing of the uterine–vaginal anastomosis. The resulting vaginal stricture was managed with surgical incision and temporary placement of a self‐expanding metallic stent. Nine months after transplantation, asymptomatic reactive tissue growth was incidentally observed during gynecologic examination beneath the distal edge of the inserted stent. Given its friable and highly vascular appearance, vaginal biopsy was performed.

Histologic examination revealed granulation tissue containing glandular structures surrounded by endometrial‐type spindle cell stroma, along with scattered hemosiderin‐laden macrophages (Figure 1). Both the glandular and stromal components demonstrated nuclear immunoreactivity for estrogen and progesterone receptor, and the stromal cells showed strong CD10 expression. These findings confirmed the diagnosis of endometriosis involving the neovaginal mucosa.

**FIGURE 1:**
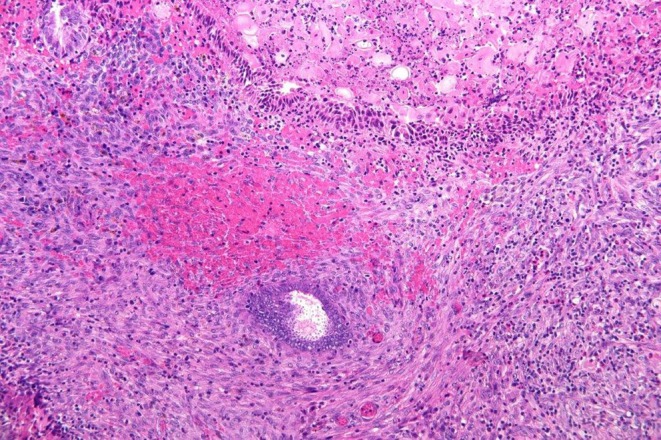
Histologic findings from the neovaginal biopsy. A glandular structure surrounded by endometrial‐type spindle cell stroma with hemorrhage (H&E, ×200).

Several unsuccessful embryo transfer attempts were subsequently performed through a canal less than 10 mm in diameter at the site of the vaginal stricture. Although the graft initially remained stable, histologic evidence of severe rejection emerged several months later and proved refractory to intensified immunosuppressive therapy. It was ultimately explanted, and histologic assessment revealed no evidence of endometriosis or neoplastic transformation.

Endometriosis involving the neovagina of a woman with MRKH syndrome is highly unusual, given the history of uterine and vaginal agenesis. A plausible explanation for the presence of ectopic endometrial tissue is iatrogenic seeding and implantation of endometrial cells from the donor uterus into the neovaginal mucosa, which had been injured by the insertion of a metallic stent. Repeated stent placement and manipulation within the neovagina may have contributed to the worsening of the stricture, as well as to the survival of ectopic cells in the altered mucosal environment. Similar iatrogenic mechanisms have been proposed in cases of endometriosis arising within abdominal wall scars following cesarean delivery or other gynecologic procedures.^7^

No endometriosis was identified in the explanted graft, reinforcing the link to local trauma from the stent rather than incidental spread. This interpretation is further supported by the facts that the uterine premenopausal donor had no history or intraoperative findings suggestive of abdominal endometriosis and the recipient had no evidence of functional endometrium in the uterine remnants.

Although not directly implicated, long‐term immunosuppression may have contributed to a permissive tissue environment, given the recognized role of immune dysregulation in endometriosis pathogenesis.^1^ However, we consider local mucosal vulnerability around the implanted metal stent a more plausible factor in the development of neovaginal endometriosis.

Although the presence of endometriotic lesions complicated atraumatic embryo transfer, the associated vaginal stricture, likely exacerbated by repeated stent placement, appears to have represented the main obstacle to achieving pregnancy.

Other contributing factors to anastomotic stricture may include a short, narrow neovagina and the use of an excessive number of absorbable sutures. These observations underscore the need for an individualized surgical approach, especially in patients with MRKH syndrome, to minimize anastomotic complications and optimize reproductive outcomes.^8^

Preventing stricture formation at the anastomotic site after transplantation is essential, as it may impede the passage of the embryo transfer catheter through the cervical canal and ultimately jeopardize the primary goal of UTx–the delivery of a healthy infant.

The UTx was performed under a research protocol approved by The Ethics Committee of the Institute for Clinical and Experimental Medicine and Thomayer Hospital (protocol no. 2044/15, NM‐15‐01). Written informed consent was obtained from the patient for participation in the study and for publication of the accompanying clinical data.

## AUTHOR CONTRIBUTIONS

Moravcova Jana, Maluskova Jana and Sticova Eva: Performed histopathologic analysis. Chmel Roman: Participated in collecting the laboratory and clinical data. Moravcova Jana, Sticova Eva and Chmel Roman: Were responsible for writing the manuscript. All authors revised and edited the draft and are in agreement with the content of the manuscript.

## FUNDING INFORMATION

No financial support was used.

## CONFLICT OF INTEREST STATEMENT

None declared.

## Data Availability

Data sharing is not applicable to this article as no new data were created or analyzed in this study.
